# A feasibility study assessing quantitative indocyanine green angiographic predictors of reconstructive complications following nipple-sparing mastectomy

**DOI:** 10.1016/j.jpra.2024.01.012

**Published:** 2024-01-26

**Authors:** J. Dalli, C.L. Nguyen, A. Jindal, J.P. Epperlein, N.P. Hardy, C. Pulitano, S. Warrier, R.A. Cahill

**Affiliations:** a UCD Centre for Precision Surgery, School of Medicine, UCD, Dublin, Ireland; b Department of Breast Surgery, Chris O'Brien Lifehouse, Camperdown, Australia; c Department of Surgery, Royal Prince Alfred Hospital, Camperdown, Australia; d Department of Surgery, The University of Sydney, Camperdown, Australia; e IBM Research Europe, Dublin, Ireland; f Department of Surgery, Mater Misericordiae University Hospital, Dublin, Ireland

**Keywords:** Oncoplastic, Indocyanine green, ICG, ICGFA, Fluorescence angiography, Immediate breast reconstruction

## Abstract

**Introduction:**

Immediate post-mastectomy breast reconstruction offers benefits; however, complications can compromise outcomes. Intraoperative indocyanine green fluorescence angiography (ICGFA) may mitigate perfusion-related complications (PRC); however, its interpretation remains subjective. Here, we examine and develop methods for ICGFA quantification, including machine learning (ML) algorithms for predicting complications.

**Methods:**

ICGFA video recordings of flap perfusion from a previous study of patients undergoing nipple-sparing mastectomy (NSM) with either immediate or staged immediate (delayed by a week due to perfusion insufficiency) reconstructions were analysed. Fluorescence intensity time series data were extracted, and perfusion parameters were interrogated for overall/regional associations with postoperative PRC. A naïve Bayes ML model was subsequently trained on a balanced data subset to predict PRC from the extracted meta-data.

**Results:**

The analysable video dataset of 157 ICGFA featured females (average age 48 years) having oncological/risk-reducing NSM with either immediate (n=90) or staged immediate (n=26) reconstruction. For those delayed, peak brightness at initial ICGFA was lower (p<0.001) and significantly improved (both quicker-onset and brighter p=0.001) one week later. The overall PRC rate in reconstructed patients (n=116) was 11.2%, with such patients demonstrating significantly dimmer (overall, p=0.018, centrally, p=0.03, and medially, p=0.04) and slower-onset (p=0.039) fluorescent peaks with shallower slopes (p=0.012) than uncomplicated patients with ICGFA. Importantly, such relevant parameters were converted into a whole field of view heatmap potentially suitable for intraoperative display. ML predicted PRC with 84.6% sensitivity and 76.9% specificity.

**Conclusion:**

Whole breast quantitative ICGFA assessment reveals statistical associations with PRC that are potentially exploitable via ML.

## Introduction

Mastectomy remains a common operation both for cancer risk reduction and treatment.^1^^,^^2^ Nipple-sparing mastectomies (NSM) with immediate breast reconstruction (IBR) offer better psychosocial and aesthetic outcomes,^3^, ^4^, ^5^ but also higher rates of complications than their alternatives (e.g., skin-sparing mastectomies (SSM) and delayed reconstructions^3^^,^^6^^,^^7^). Nipple and skin malperfusion, for instance, can lead to necrotic complication rates between 3% and 20%.^3^^,^^8^ Malperfusion also contributes to wound management problems, psychological distress, poor cosmesis, infection, prosthesis loss, reoperations, adjuvant therapy delay and further costs.^9^

Intraoperative perfusion assessment with indocyanine green fluorescence angiography (ICGFA) using near-infrared (NIR) cameras has been demonstrated to cost-effectively diminish postoperative complications in autologous and implant-based oncoplastic reconstructions.^10^^,^^11^ A recent study also investigated its routine use in selecting patients who may benefit from a temporary delay in performing reconstruction (termed staged immediate breast reconstruction, SIBR) by measuring fluorescence intensity from still images^11^ in decision-making albeit with modest sensitivity and specificity regarding complication prediction (62.5% and 69.5%, respectively).^12^, ^13^, ^14^

Current practice of visual ICGFA interpretation across surgical specialities has, however, been demonstrated to be subjective and experiential.^15^ The dynamic inflow/outflow perfusion signal can be quantified from NIR videos into time series curves from selected regions of interest (ROI).^16^ Such quantitative (Q)-ICGFA patterns have been associated with postoperative surgical complications, and these statistics have also supported the development of predictive machine learning (ML) algorithms.^17^^,^^18^

Here, we seek to develop the case for ICGFA beyond both observer interpretation and still image sampling to computational full curve dynamic assessment using videos from a series of patients undergoing flap ICGFA following NSM with subsequent IBR or SIBR in a clinical trial. We investigate the clinical relevance of such fluorescence metrics in relation to the decision to delay and the occurrence of perfusion-related complications (PRC). Furthermore, we determine the potential for such fluorescence metrics to be visually displayed as heatmaps and to train a ML model for complication prediction.

## Method

**Figure 1:**
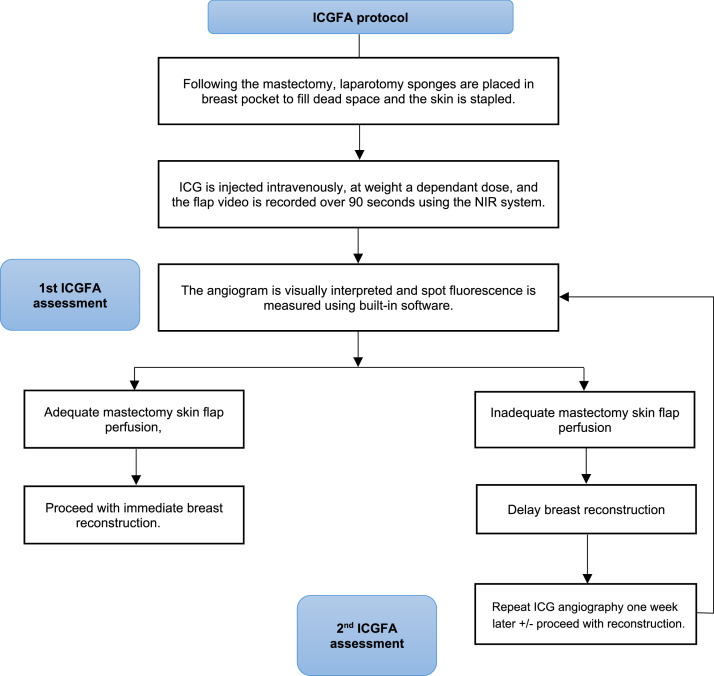
Clinical pathway from the previous study protocol^12^. ICG denotes indocyanine green and NIR denotes near infrared.

Patients: In a previous single-centre study (Protocol X17-0359 HREC/17/RPAH/542, see Figure 1 for operative workflow)^12^, 213 patients undergoing NSM were subsequently assessed via ICGFA. The standardised mastectomy technique involved preoperative antibiotics, inframammary incision, electrocautery dissection superficial to the anterior mammary fascia, and no intraoperative local anaesthesia/epinephrine injection. The mastectomy pocket was filled with a laparotomy sponge, and the incision was temporarily closed with staples.

Once the NIR camera (SPY Q Elite Fluorescence Imaging System, Stryker, USA) was positioned using the 2-dot laser-guided distance marker, ICG (Infracyanine®, 25 mg/10mL, SERB, Paris, France) was administered intravenously at a weight-dependent dosage. The mastectomy flap ICGFA including the skin and nipple-areolar complex (NAC) were observed and recorded at a rate of 30 frames per second over 90 seconds after fluorescence was first detected. Dynamic ICGFA appearances were visually interpreted in real-time, including measuring fluorescence from still images using the built-in software of the SPY Q system from a single frame at 90 seconds. IBR proceeded if perfusion was deemed sufficient. If the flap demonstrated poor perfusion patterns by visual NIR interpretation and an absolute fluorescence intensity of less than 14 units^13^, the wound was closed without attempting reconstruction. Drains were routinely inserted.

**Figure 2:**
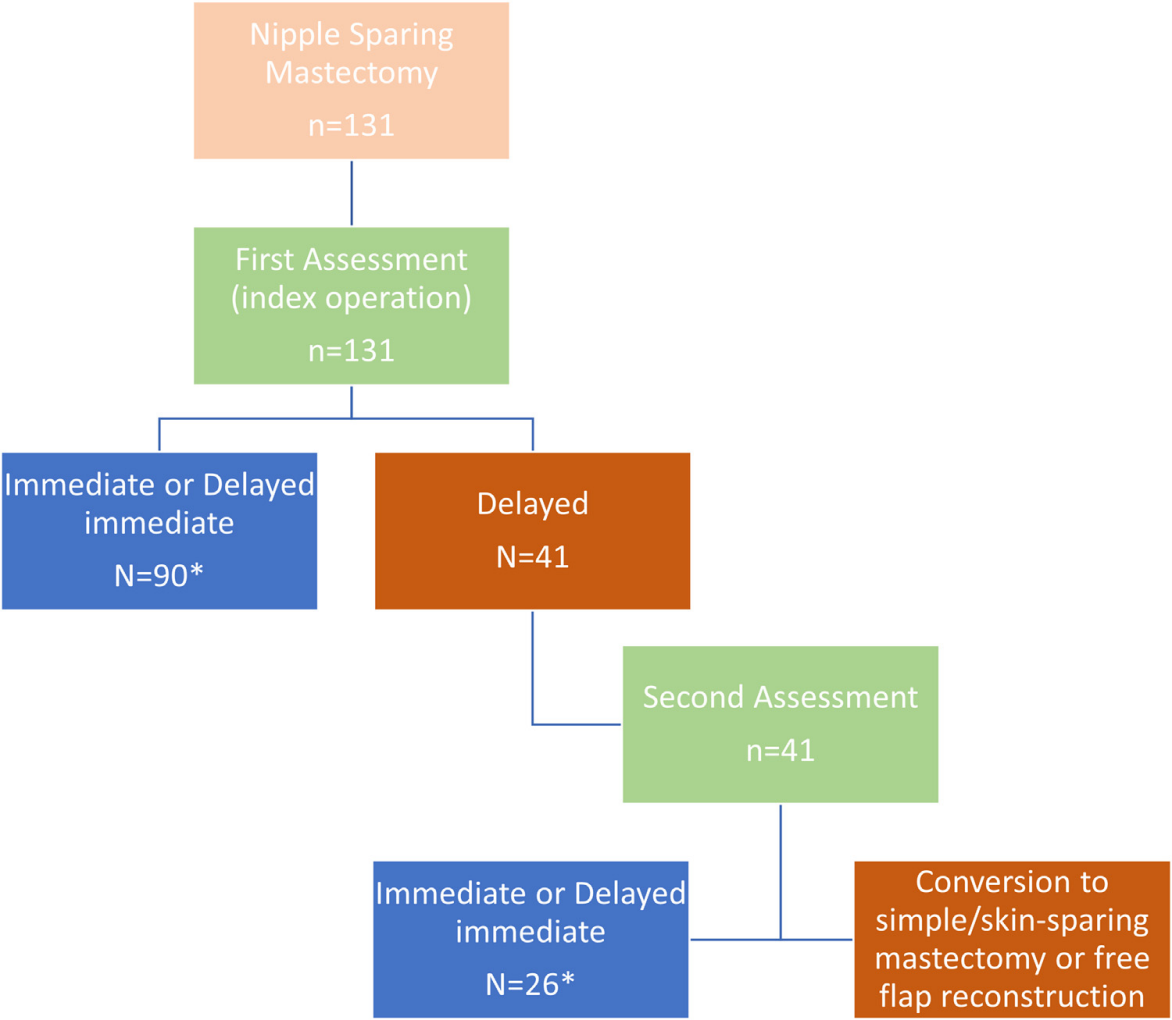
Flow chart showing the analysable video dataset. 131 patients underwent ICG assessment following NSM. * Denotes videos analysed with quantitative fluorescence (n=116) and from which the balanced data subset for ML training was obtained (n=26). These included cases following NSM where the first (n=90) or second (n=26) assessment supported immediate or delayed immediate (tissue expander) reconstruction (blue boxes). Reconstruction was deemed unsafe at the first assessment for n=41, and these were only investigated with quantitative fluorescence and not included in ML training. Green boxes denote intraoperative assessment, with the second assessment taking place one week following the deferral of reconstruction at the first operation.

For those not reconstructed at this procedure, a similar assessment was then performed a week later to determine if perfusion was sufficient or converted to alternative procedures (e.g., SSM) if insufficient (see Figures 1 and 2).

Reconstructions, whether immediate or staged, were carried out via the pre-pectoral technique with a tissue expander or direct-to-implant (using gel prosthesis and ADM: acellular dermal matrix or mesh^19^). Patient demographics and operative complications up to 90 days were recorded, including partial or full-thickness necrosis, wound dehiscence, haematomas, seromas, infections, implant loss, and reoperations. PRC grouped any partial/full-thickness necrosis, wound dehiscence, infection, and implant loss into a yes/no categorisation (including if the patient suffered more than one such complication).

**Figure 3:**
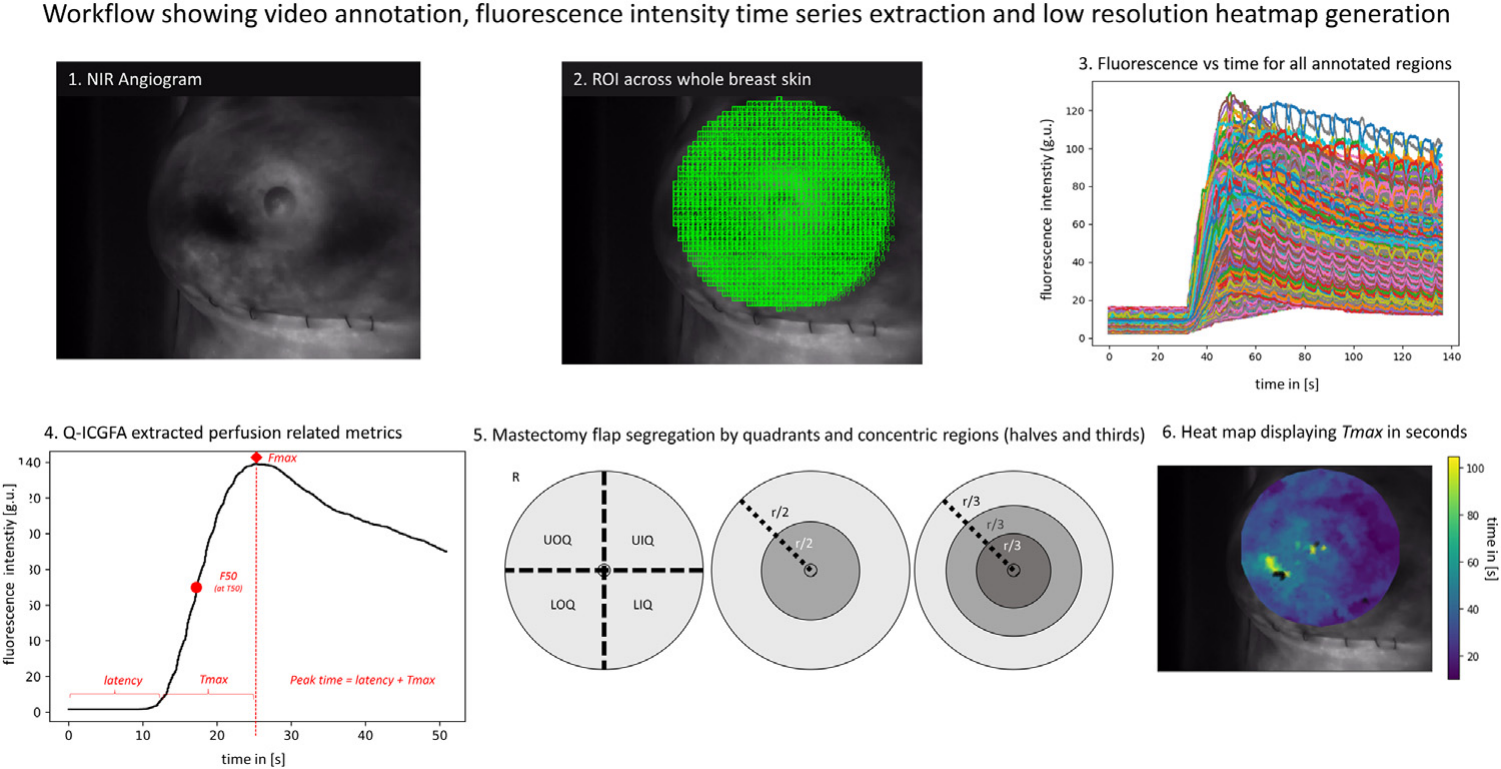
Q-ICGFA workflow (index angiogram for a 58-year-old lady following oncoplastic NSM, delayed and underwent SIBR). Panel 1 shows the angiogram, with breast pocket temporarily closed and surgical gauze in the cavity. Panel 2 shows the mastectomy flap annotated with 14×14 boxes, and the fluorescence time series extracted per box are shown in Panel 3. Panel 4 shows extracted perfusion-related metrics (see Methods). Panel 5 shows mastectomy flap region segregation by quadrants and concentric rings (r: radius bisected or divided in 3). R denotes right breast, UOQ upper outer, UIQ upper inner, LOQ lower outer, and LIQ lower inner quadrants. Panel 6 shows a low-resolution heat map with x and y-axis showing coordinates and a colour scale representing time in seconds.

Q-ICGFA: In this study, ICGFA recordings from the above patient cohort were analysed quantitatively (see Figure 3 for quantification workflow). To accomplish this, the dataset was first cleaned through visual inspection of videos and those with excessive movement, instrument intrusion, bleeding, fluorescence saturation, or missed inflow were excluded from subsequent analysis. Using previously described software^16^, fluorescence intensity time series were plotted from the mastectomy flap ICGFA (annotated as a single ROI) that was computationally divided into squares of 14×14 pixels. Previously reported arterial inflow milestones^16^^,^^17^ relating to fluorescence brightness (*F*) and curve chronology (*T*) were extracted directly from the time series curves (denoted by *n*) or following curve smoothing via a Savitzky–Golay filter to compensate for signal noise and fine movements.

*Latency* was defined as the period between ICG injection and inflow initiation (in seconds, see Figure 3). *F*_max_/*nF*_max_ denotes peak brightness, and the time from the beginning of the video to the attainment of maximum brightness was denoted as *peak time* (this includes *latency*). *T*_max_/*nT*_max_ is the time between the end of *latency* and *F*_max_*,* and its upward gradient is denoted as *upslope. T_50_* denotes the time required to achieve half of *F*_max_, with the fluorescence at this point being *F_50_* and the gradient prior to this point *upslope_50_*.

For further analysis, three strategies were tested to segregate the mastectomy flap region based on either quadrants or rings (see Figure 3) in order to establish relationships between clinical outcome predictability and regional quantitative metrics.

High-resolution heatmaps were generated to demonstrate the feasibility of capturing dynamic perfusion parameters from every pixel on ICGFA videos into summarising images, visually presenting data values as colours (n=6, see Figure 3).

Demographics for all patients whose recordings were analysed in this study were compared based on the timing of reconstruction and clinical outcomes, including PRC.

Statistical analysis: Using SPSS Version 27 (IBM, USA), normality was assessed with the Shapiro–Wilk test. Independent and dependent comparisons were tested using the Mann–Whitney U and Wilcoxon-signed rank, respectively. Frequencies were compared with Fisher's exact test. Significance was ascribed when *p* < 0.05.

Metrics measuring fluorescence for regions or the whole mastectomy flap from ICGFA recordings of patients who underwent reconstruction at index operation were compared to those whose reconstruction was delayed. In addition, fluorescence milestone data from ICGFA videos at the second assessment were compared to the same patients at index operation. Furthermore, fluorescence metrics for all reconstructions (IBR and SIBR) were statistically compared for association with PRC, providing the basis for ML development.

ML: Using MATLAB® R2022b (MathWorks®, USA), the most important fluorescence metrics and mastectomy flap region in predicting PRCs were identified and ranked using -log(p) of the Kruskal–Wallis test.^20^ We used the Classification Learner application to train multiple models (such as naïve Bayes, ensemble, decision trees, and *k*-nearest neighbour) to predict PRCs in a binary outcome fashion. We report results for the best-performing model, which was determined using the area under the ROC (receiver operating characteristic) curve (AUC), sensitivity, specificity, and positive and negative predictive values (PPV and NPV). It is important to note that the models were only trained on a subset of the data that included an equal number of patients suffering PRCs and those not suffering PRCs. This was necessary because the actual data was heavily unbalanced in favour of patients not suffering PRCs, and we found that the models would not deviate from the high accuracy that resulted from simply predicting non-PRC for all cases.

## Results

Data collection: Of 299 ICGFA recordings, 142 were excluded from subsequent analysis (114 and 28 patients at first and second assessment, respectively). In 118, arterial inflow was missed (n=105 were bilateral cases assessing both sides within the same angiogram, resulting in insufficient recording time in one or both breasts). The remaining 24 were excluded because of excessive camera movement (n=4), signal saturation (n=13, see Figure 4.4), instrument/hand intrusion (n=5), and bleeding (n=2).

**Figure 4:**
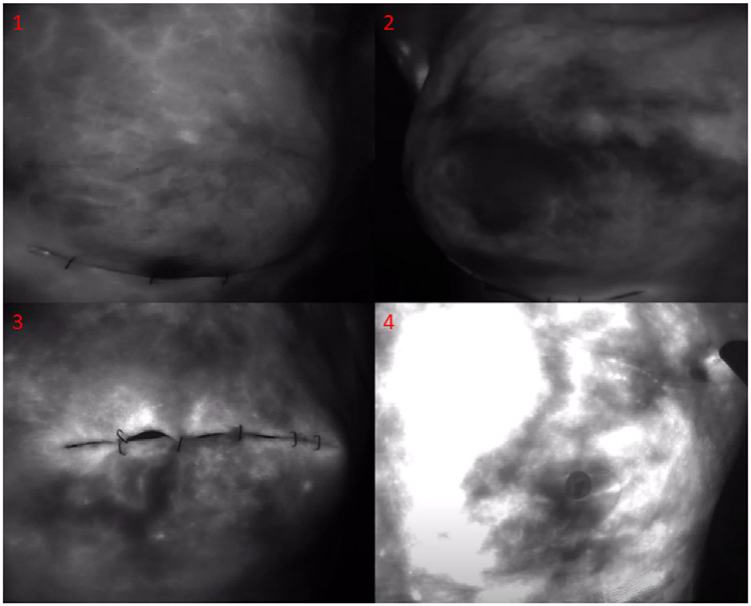
Single frames from ICGFA for illustrative cases. Top left (1) shows the left skin flap of a 60-year-old following NSM for T2N0 invasive ductal carcinoma followed by immediate tissue-expander reconstruction with an uneventful recovery. Top right (2) shows the right mastectomy flap of a 43-year-old ex-smoker who underwent IBR following unilateral NSM and SNLB for T2N0 lobular carcinoma and subsequently suffered PRC and implant loss. The bottom images show angiograms from excluded cases. Skin-sparing mastectomies (bottom left, 3) were not included in the study as the central, temporarily closed wound presented gaps that would not fluoresce, potentially misguiding Q-ICGFA. Saturated ICGFA (bottom right, 4) were excluded from statistical and machine learning analysis. This angiogram was acquired at the second assessment (one week following prophylactic NSM at index procedure). This lady received tissue expanders and recovered uneventfully.

Of the 157 included ICGFA recordings, 131 were from the first assessment, and 26 from the delayed second assessment (see Figure 2). Of the former, 90 were of patients who had IBR, while 41 were patients who had delayed reconstruction at index operation (23 of these patients also had processable videos from their second assessment). Demographics and indications for surgery of the patients included in this study are shown in Table 1. Notably, a greater proportion of those having bilateral surgery and simultaneous mastopexies underwent IBR. The first intraoperative assessment identified insufficient perfusion in all those with a history of local radiotherapy (n=3).

**Table 1:** Clinical data and case demographics of the patients included in this study (at index procedure) including whether they had immediate reconstruction, or this was delayed. Figures are presented as frequencies with percentages in brackets. S.d. denotes standard deviation. DCIS denotes ductal carcinoma in situ. *P* values for the Mann–Whitney U test for continuous variables (only age) and Fischer exact test for comparisons of frequencies. Significant results are marked with*.

Clinical data of patients at index procedure					
	Criteria	Total	Reconstruction	Delayed	*p* value
Case demographics	*N*	131	90	41	
	Mean age in years	47.8 ± 10.2	47.1 ± 10.6	49.4 ± 9	0.091
	Right: Left	77:54	52:38	25:16	0.849
	Bilateral	67 (52.7%)	55 (61.1%)	14 (34.2%)	0.005*
	Invasive cancer	79 (60.3%)	51 (56.7%)	28 (68.3%)	0.250
	T1: T2 or greater	21 (16%):58 (44.3%)	17 (18.9%):34 (37.8%)	4 (9.8%):24 (58.5%)	0.109
	N0: N1 or greater	56 (42.7%):23 (17.6%)	37 (41.1%):14 (15.6%)	19 (46.3%):9 (22%)	0.796
	DCIS	30 (22.9%)	20 (22.2%)	10 (24.4%)	0.824
	Risk reducing	28 (21.4%)	20 (22.2%)	8 (19.5%)	0.821
	Tissue expander used	62 (47.3%)	62 (72.2%)	n/a	n/a
	Direct to implant	23 (17.6%)	25 (27.8%)	n/a	n/a
	Mastopexy	5 (3.8%)	5 (5.56%)	0.0%	0.008*
Co-morbidities	Smoking	12 (9.2%)	7 (7.78%)	5 (12.2%)	0.515
	Diabetes mellitus	1 (0.8%)	1 (1.1%)	0	1.000
	Hypertension	2 (1.5%)	2 (2.2%)	0	1.000
	History of radiotherapy	3 (2.3%)	0	3 (7.3%)	0.029*

Four patients required excision of clinically determined necrotic NAC and skin at second assessment and were excluded (n=2 received tissue expander, n=2 not reconstructed).

On assessing all those undergoing reconstructions (IBR and SIBR n =116), those suffering from PRC (11.2% n=13, Table 2) had similar ages, case demographics, and co-morbidities compared to those without complications. All complications except one (NAC necrosis, 0.8%) were related to the skin flaps.

**Table 2:** Demographics and clinical outcomes of patients undergoing breast reconstruction (IBR and SIBR) in relation to perfusion-related complications (PRC) showing figures as frequencies with percentages in brackets. PRC is a binary metric of patients suffering necrosis, infection, dehiscence, and/or implant loss. S.d. denotes standard deviation. DCIS denotes ductal carcinoma in situ. *P* values for the Mann–Whitney U test for continuous variables (only age) and Fischer exact test for comparisons of frequencies. Significant results are marked with *.

Demographics and clinical outcomes of patients undergoing breast reconstruction (IBR and SIBR) in relation to PRC					
		All	No PRC	PRC	*p* value
Case demographics	*N*	116	103	13	
	Mean age in years ± s.d.	47.8 ± 10	47.7 ± 10.3	47.9 ± 8.2	0.913
	Right: Left	68:48	59:44	9:4	0.554
	Bilateral	55(47.4%)	48 (46.6%)	7 (53.8%)	0.770
	Invasive cancer	67 (57.8%)	57 (55.3%)	10 (76.9%)	0.233
	T1: T2 or greater	19 (16.4%): 48 (41.4%)	18 (17.5%):39 (37.9%)	1 (7.7%) :9 (69.2%)	0.260
	N0: N1 or greater	49 (42.2%) :18 (15.5%)	42 (40.8%) :15 (14.6%)	7 (5.4%):3 (23.1%)	1.000
	DCIS	26 (22.4%)	23 (22.3%)	3 (23.1%)	1.000
	Risk reducing	27 (23.3%)	26 (25.2%)	1 (7.7%)	0.294
	Implant delayed	26 (22.4%)	24 (23.3%)	2 (15.4%)	0.730
	Expander	88 (75.9%)	80 (77.7%)	8 (61.5%)	0.299
	Direct to implant	28 (24.1%)	23 (22.3%)	5 (38.5%)	0.299
	Mastopexy	7 (6.0%)	7 (6.8%)	0	1.000
Co-morbidities	Smoking	9 (7.8%)	7 (6.8%)	2 (15.4%)	0.265
	Diabetes mellitus	1 (0.9%)	1 (1.0%)	0	1.000
	Hypertension	3 (2.6%)	3 (2.9%)	0	1.000
	History of radiotherapy	1 (0.86%)	1 (0.97%)	0	0.888
Perfusion-related complications (PRC)	Necrosis (nipple or skin flap)	2 (1.7%)	0	11 (84.6%)	n/a
	Infection	7 (6.0%)	0	7 (53.8%)	n/a
	Dehiscence	2 (1.7%)	0	2 (15.4%)	n/a
	Implant loss	4 (3.5%)	0	4 (30.8%)	n/a
	Total number of patients suffering a PRC	13 (11.2%)	0	13 (100%)	n/a
Other complications	Haematoma	3 (2.6%)	1 (1.0%)	2 (15.4%)	0.033*
	Seroma	7 (6.0%)	3 (2.9%)	4 (30.8%)	0.003*
	Reoperation	7 (6.0%)	0	7 (53.8%)	<0.001*

Patients with PRC also exhibited more non-PRC complications (haematomas and seromas) and an overall increased reoperation rate. There were no significant differences in PRC proportions in those undergoing IBR following the index procedure (12.2% n=11 from n=90) versus those reconstructed a week later (7.8% n=2 from n=26).

### Q-ICGFA

*Index operation:* Broadly, time-based metrics did not compute significant differences across the whole breast or on a regional level. On direct comparison, the ICGFA was brighter for IBR versus delayed cases across the whole breast (*nF_max_* p<0.001) and on a regional level (see Table 3) during the first assessment. Time to peak was brisker only in the lower medial quadrant (*nT_max_* p=0.037) (see supplementary).

**Table 3:** This table shows the mean extracted quantitative fluorescence metrics relating to intensity in g.u. and chronology in seconds for the annotated breast, divided by quadrants and concentric regions. Features were extracted directly from the time series (n) or with a curve detection algorithm following mathematical smoothing (see supplementary data for further metrics). Fluorescence metrics for those undergoing IBR were compared to staged immediate cases delayed at the index procedure via the Mann–Whitney U test. Significant results are marked with *.

Fluorescence metrics at the first (index) procedure for IBR versus delayed cases															
Quadrant	Whole Breast			Lower Lateral			Lower Medial			Upper Lateral			Upper Medial		
	IBR	Delay	p	IBR	Delay	p	IBR	Delay	p	IBR	Delay	p	IBR	Delay	p
latency (s)	17.80 ± 9.80	17.31 ± 6.99	0.966	18.91 ± 10.11	18.24 ± 7.52	0.843	18.52 ± 9.64	17.76 ± 7.32	0.550	18.71 ± 10.09	17.99 ± 7.44	0.699	18.60 ± 10.18	17.79 ± 7.25	0.781
peak time (s)	52.16 ± 19.95	46.66 ± 14.72	0.341	55.98 ± 19.68	57.90 ± 16.86	0.480	53.74 ± 23.52	56.41 ± 20.22	0.356	51.13 ± 21.20	51.34 ± 17.97	0.702	53.74 ± 24.18	51.76 ± 24.90	0.619
Fmax (g.u.)	73.61 ± 32.21	57.60 ± 23.79	0.056	69.17 ± 29.43	51.97 ± 28.52	0.020*	53.74 ± 23.52	56.40 ± 24.84	0.009*	76.77 ± 37.47	64.96 ± 37.71	0.145	84.59 ± 43.15	71.02 ± 34.64	0.291
Tmax (s)	34.43 ± 17.01	29.65 ± 12.83	0.267	36.08 ± 16.78	40.12 ± 19.72	0.425	34.49 ± 20.71	40.26 ± 21.13	0.168	32.87 ± 18.69	33.65 ± 17.40	0.783	35.20 ± 20.48	33.99 ± 22.70	0.581
Upslope (g.u./s)	2.96 ± 2.83	2.33 ± 1.68	0.621	2.52 ± 2.28	1.88 ± 1.77	0.110	3.51 ± 3.41	2.03 ± 1.68	0.035*	3.40 ± 3.40	2.71 ± 2.29	0.449	3.75 ± 3.94	3.42 ± 3.90	0.641
nTmax (s)	42.00 ± 23.35	49.49 ± 31.05	0.357	46.29 ± 24.30	55.80 ± 30.07	0.119	43.34 ± 25.51	53.98 ± 28.67	0.037*	40.58 ± 23.74	48.52 ± 31.57	0.316	38.90 ± 23.09	49.98 ± 34.55	0.208
nFmax (g.u.)	71.12 ± 31.18	49.60 ± 23.75	<0.001*	64.91 ± 29.19	42.10 ± 26.75	<0.001*	75.59 ± 36.35	48.38 ± 25.96	<0.001*	72.12 ± 35.94	56.11 ± 35.45	0.009*	82.49 ± 42.40	61.88 ± 35.06	0.013*



Concentric Zones	Inner half			Outer half			Inner third			Middle third			Outer third		
	IBR	Delay	p	IBR	Delay	p	IBR	Delay	p	IBR	Delay	p	IBR	Delay	p
latency (s)	18.46 ± 10.05	17.81 ± 7.58	0.762	17.71 ± 9.61	17.10 ± 6.84	0.829	19.55 ± 10.21	19.36 ± 7.22	0.909	17.78 ± 9.82	17.39 ± 7.00	0.943	17.62 ± 9.49	16.90 ± 6.79	0.715
peak time(s)	60.09 ± 26.03	54.05 ± 20.73	0.456	48.95 ± 19.45	50.36 ± 21.23	0.773	64.15 ± 27.02	65.04 ± 23.54	0.574	50.92 ± 19.46	46.18 ± 16.47	0.328	47.55 ± 18.95	49.42 ± 18.93	0.567
Fmax (g.u.)	83.19 ± 33.71	58.43 ± 24.45	0.003*	68.24 ± 32.24	54.64 ± 27.76	0.060	87.94 ± 38.23	50.51 ± 32.81	<0.001*	74.37 ± 33.61	59.74 ± 24.81	0.129	63.22 ± 30.16	52.06 ± 28.38	0.054
Tmax (s)	40.96 ± 22.09	36.11 ± 20.38	0.285	31.88 ± 17.35	33.68 ± 20.29	0.886	43.56 ± 22.56	46.26 ± 24.11	0.586	33.40 ± 17.04	28.48 ± 13.38	0.232	29.95 ± 15.70	32.83 ± 18.02	0.531
Upslope (g.u./s)	2.89 ± 2.57	2.05 ± 1.50	0.331	3.04 ± 3.06	2.39 ± 2.07	0.294	2.76 ± 2.35	1.49 ± 1.43	0.005*	3.07 ± 2.99	2.57 ± 1.82	0.856	2.94 ± 2.88	2.35 ± 2.24	0.219
nTmax (s)	47.57 ± 24.78	55.61 ± 31.03	0.200	35.10 ± 20.40	42.35 ± 28.21	0.324	51.55 ± 25.37	58.06 ± 28.21	0.189	38.80 ± 21.73	47.34 ± 31.51	0.348	33.39 ± 19.80	38.10 ± 24.19	0.374
nFmax (g.u.)	79.19 ± 33.24	49.75 ± 25.44	<0.001*	66.36 ± 31.99	51.73 ± 26.54	0.017*	84.08 ± 35.98	49.87 ± 29.80	<0.001*	71.51 ± 32.86	51.35 ± 25.17	0.002*	61.43 ± 30.02	51.05 ± 26.78	0.055

*Second assessment:* In patients who underwent a repeat assessment after delay, ICGFA were brighter and brisker across the whole breast when compared to the first assessment. Fluorescence intensity was significantly higher (*F_max_* p=0.001, *nF_max_* p<0.001), and time-based metrics were quicker (*peak time* p=0.039 and *nT_max_* p=<0.001), with also regional improvements in *T_max_* evident at the second assessment (Table 4 and supplementary).

**Table 4:** This table shows the mean extracted quantitative fluorescence metrics relating to intensity in g.u. and chronology in seconds for the annotated breast, divided by quadrants and concentric regions. Fluorescence metrics for those patients who were delayed at the first (index) assessment versus the same cases at the second assessment a week later via Wilcoxon-signed rank. Features were extracted directly from the time series (*n*) or with a curve detection algorithm following mathematical smoothing (see supplementary data for further metrics). Significant results are marked with *.

Fluorescence metrics for delayed cases at first (index) assessment versus second assessment a week later															
Quadrant	Whole Breast			Lower Lateral			Lower Medial			Upper Lateral			Upper Medial		
	First	Second	p	First	Second	p	First	Second	p	First	Second	p	First	Second	p
latency (s)	18.22 ± 6.79	15.22 ± 8.71	0.316	19.63 ± 7.27	15.59 ± 8.70	0.136	19.30 ± 7.21	15.43 ± 8.92	0.073	18.82 ± 7.43	15.49 ± 8.93	0.287	18.98 ± 6.81	14.96 ± 8.79	0.055
peak time (s)	46.84 ± 12.65	35.87 ± 12.89	0.039*	57.66 ± 18.51	38.42 ± 13.91	0.005*	57.62 ± 19.41	37.09 ± 12.09	0.023*	51.09 ± 18.27	36.18 ± 12.23	0.005*	51.22 ± 27.69	34.33 ± 10.45	0.047*
Fmax (g.u.)	55.59 ± 17.32	109.28 ± 40.27	0.001*	48.68 ± 23.46	100.47 ± 38.74	0.008*	58.06 ± 22.89	108.44 ± 41.81	0.008*	60.12 ± 28.79	113.25 ± 42.17	0.006*	72.45 ± 24.43	119.11 ± 46.99	0.005*
Tmax (s)	29.56 ± 12.16	21.43 ± 7.38	0.101	39.99 ± 22.65	23.64 ± 9.63	0.041*	40.19 ± 23.18	22.24 ± 8.00	0.034*	33.87 ± 17.89	21.14 ± 6.99	0.027*	32.34 ± 24.86	19.45 ± 5.51	0.078
Upslope (g.u./s)	2.12 ± 1.07	5.64 ± 2.82	0.005*	1.81 ± 1.59	5.10 ± 3.02	0.010*	2.09 ± 1.63	5.34 ± 2.55	0.012*	2.57 ± 2.11	5.95 ± 3.17	0.002*	3.15 ± 1.97	6.57 ± 3.48	0.017*
nTmax (s)	47.77 ± 29.35	23.77 ± 11.79	<0.001*	55.32 ± 31.83	25.74 ± 13.26	0.002*	55.83 ± 27.49	25.69 ± 13.03	<0.001*	47.86 ± 30.39	25.17 ± 12.44	0.004*	51.49 ± 35.91	23.49 ± 13.06	0.004*
nFmax (g.u.)	49.01 ± 18.59	104.07 ± 39.19	<0.001*	43.11 ± 22.42	95.85 ± 37.58	<0.001*	48.86 ± 25.46	105.27 ± 41.84	<0.001*	54.98 ± 28.66	106.15 ± 43.94	<0.001*	60.70 ± 30.75	115.97 ± 46.66	<0.001*



Concentric Zones	Inner half			Outer half			Inner third			Middle third			Outer third		
	First	Second	p	First	Second	p	First	Second	p	First	Second	p	First	Second	p
latency (s)	18.98 ± 7.71	15.39 ± 8.65	0.274	18.13 ± 6.48	15.18 ± 8.72	0.308	20.78 ± 6.62	15.41 ± 8.67	0.046*	18.15 ± 6.92	15.00 ± 8.95	0.301	18.13 ± 6.53	15.19 ± 8.75	0.330
peak time(s)	56.57 ± 18.29	37.42 ± 12.93	0.026*	45.79 ± 16.61	35.28 ± 12.20	0.011*	68.53 ± 20.55	41.03 ± 14.49	0.006*	46.92 ± 16.32	35.32 ± 12.42	0.028*	48.15 ± 18.39	35.25 ± 11.12	0.023*
Fmax (g.u.)	53.49 ± 18.18	113.37 ± 42.40	0.003*	54.22 ± 18.82	105.05 ± 40.92	<0.001*	47.99 ± 24.28	116.66 ± 45.52	0.003*	57.51 ± 18.12	110.26 ± 41.20	<0.001*	49.28 ± 20.67	98.64 ± 38.99	<0.001*
Tmax (s)	37.72 ± 18.61	22.75 ± 7.78	0.033*	28.11 ± 15.53	20.51 ± 7.13	0.079	49.59 ± 22.27	25.69 ± 8.93	0.006*	28.70 ± 14.09	21.15 ± 7.08	0.064	30.80 ± 18.28	20.27 ± 6.43	0.052
Upslope (g.u./s)	1.71 ± 1.03	5.61 ± 3.01	0.006*	2.40 ± 1.41	5.74 ± 3.10	0.001*	1.17 ± 0.85	5.29 ± 3.25	0.002*	2.35 ± 1.28	5.78 ± 2.96	0.004*	2.13 ± 1.38	5.42 ± 3.08	<0.001*
nTmax (s)	55.92 ± 28.66	25.09 ± 12.39	<0.001*	41.06 ± 30.05	22.94 ± 11.95	0.011*	57.66 ± 23.04	27.30 ± 12.70	<0.001*	45.69 ± 30.79	23.53 ± 11.68	0.003*	37.89 ± 26.15	21.11 ± 7.45	0.010*
nFmax (g.u.)	50.57 ± 21.29	108.60 ± 40.89	<0.001*	50.09 ± 19.49	101.05 ± 40.26	<0.001*	51.31 ± 25.24	112.60 ± 43.44	<0.001*	50.81 ± 19.87	104.42 ± 40.33	<0.001*	48.61 ± 19.39	96.26 ± 39.77	<0.001*

*Reconstructions PRC versus no PRC associations:* Those with reconstruction (whether IBR or SIBR) without PRC demonstrated brighter (*F_max_* p=0.018 and *nF_max_* p= 0.035), brisker angiograms (*T_max_* p=0.039), with sharper inflow (*upslope* p=0.012) across the entire flap than those who developed PRC (see Table 5 and supplementary). The medial quadrants (upper and lower) of those with PRC showed dimmer angiograms (*nF_max_* p=0.035 and p=0.039). Those with PRC also had dimmer (*nF_max_* inner half p=0.030 and inner third *nF_max_* p=0.036) and slower (*T_max_* p=0.042 and p=0.022, respectively) ICGFA appearances centrally. Additionally, a delayed *peak time* in the outer half and third of the skin was associated with complications (p=0.024 and p=0.020, respectively). Prolonged *latency* across all quadrants was also regionally associated with PRC (p=0.014 to 0.039).

**Table 5:** This table shows the mean extracted quantitative fluorescence metrics relating to intensity in g.u. and chronology in seconds for the annotated breast, divided by quadrants and concentric regions. Fluorescence metrics for patients who underwent reconstruction (IBR and SIBR) and suffered PRC were compared to those who did not via the Mann–Whitney U test. Features were extracted directly from the time series (*n*) or with a curve detection algorithm following mathematical smoothing (see supplementary data for further metrics). Significant results are marked with *.

Fluorescence metrics for all patients who underwent reconstructions (IBR and SIBR) for those who did and did not develop PRC															
Quadrant	Whole Breast			Lower Lateral			Lower Medial			Upper Lateral			Upper Medial		
	No PRC	PRC	p	No PRC	PRC	p	No PRC	PRC	p	No PRC	PRC	p	No PRC	PRC	p
latency (s)	16.59 ± 8.49	20.76 ± 11.31	0.065	17.55 ± 9.03	21.70 ± 10.34	0.031*	17.06 ± 8.58	21.89 ± 10.72	0.014*	17.29 ± 8.89	21.76 ± 11.13	0.039*	17.12 ± 8.96	21.31 ± 11.00	0.036*
Peak time (s)	45.87 ± 17.90	58.84 ± 18.61	0.004*	50.74 ± 18.41	61.86 ± 22.71	0.071	49.47 ± 22.01	60.99 ± 17.54	0.018*	46.89 ± 19.35	58.25 ± 23.42	0.058	48.68 ± 23.31	60.48 ± 26.79	0.040*
Fmax (g.u.)	79.10 ± 37.47	59.38 ± 25.47	0.018*	73.56 ± 37.67	59.76 ± 22.39	0.214	82.80 ± 40.33	62.45 ± 27.82	0.065	83.01 ± 42.46	64.20 ± 33.19	0.070	89.72 ± 46.80	70.69 ± 32.19	0.113
Tmax (s)	29.31 ± 14.01	39.69 ± 20.47	0.039*	32.99 ± 16.13	40.34 ± 23.19	0.310	32.51 ± 19.79	38.91 ± 17.94	0.129	30.05 ± 17.05	37.51 ± 24.37	0.351	31.62 ± 20.01	39.38 ± 27.07	0.204
Upslope (g.u./s)	3.57 ± 3.14	2.06 ± 2.03	0.012*	3.16 ± 3.06	2.15 ± 2.33	0.185	3.69 ± 3.20	2.23 ± 2.06	0.054	3.94 ± 3.64	2.82 ± 3.08	0.094	4.42 ± 4.34	2.72 ± 2.60	0.108
nTmax (s)	40.11 ± 26.29	50.12 ± 26.79	0.068	44.63 ± 26.69	52.66 ± 30.29	0.246	41.64 ± 25.67	52.78 ± 27.80	0.086	38.34 ± 24.64	52.19 ± 32.31	0.087	38.13 ± 26.66	50.14 ± 31.85	0.080
nFmax (g.u.)	72.57 ± 36.67	56.71 ± 24.57	0.035*	64.73 ± 36.65	56.60 ± 20.85	0.533	75.03 ± 40.09	55.20 ± 27.18	0.033*	75.23 ± 41.60	61.63 ± 30.35	0.150	85.28 ± 46.32	63.27 ± 33.10	0.039*



Concentric Zones	Inner half			Outer half			Inner third			Middle third			Outer third		
	No PRC	PRC	p	No PRC	PRC	p	No PRC	PRC	p	No PRC	PRC	p	No PRC	PRC	p
latency (s)	17.15 ± 8.86	21.09 ± 11.23	0.102	16.50 ± 8.39	20.42 ± 10.99	0.062	18.19 ± 9.09	21.99 ± 10.59	0.080	16.59 ± 8.58	20.54 ± 11.26	0.092	16.36 ± 8.28	20.66 ± 10.89	0.038*
peak time (s)	52.41 ± 23.88	70.02 ± 25.51	0.006*	45.66 ± 19.32	54.74 ± 19.14	0.024*	58.08 ± 26.12	75.15 ± 25.76	0.008*	45.43 ± 17.97	56.14 ± 19.08	0.016*	44.55 ± 18.13	53.85 ± 18.35	0.020*
Fmax (g.u.)	85.62 ± 40.02	66.25 ± 22.33	0.080	72.24 ± 37.43	58.68 ± 29.81	0.078	87.30 ± 46.19	61.75 ± 25.73	0.037*	79.43 ± 38.23	62.32 ± 28.20	0.049*	67.69 ± 35.74	55.35 ± 27.58	0.100
Tmax (s)	35.10 ± 20.61	48.77 ± 25.87	0.042*	29.57 ± 17.10	35.78 ± 20.17	0.136	39.66 ± 23.36	52.70 ± 23.84	0.022*	28.91 ± 14.47	36.45 ± 20.30	0.110	28.16 ± 15.18	34.61 ± 19.26	0.122
Upslope (g.u./s)	3.46 ± 3.10	1.91 ± 1.74	0.031*	3.52 ± 3.29	2.29 ± 2.43	0.051	3.22 ± 3.04	1.46 ± 1.21	0.010*	3.66 ± 3.26	2.31 ± 2.27	0.036*	3.39 ± 3.13	2.26 ± 2.45	0.053
nTmax (s)	44.68 ± 27.35	57.79 ± 27.14	0.033*	34.04 ± 22.85	44.44 ± 28.38	0.079	48.10 ± 27.20	61.25 ± 24.29	0.021*	37.85 ± 25.16	46.53 ± 28.50	0.131	31.30 ± 19.56	43.43 ± 28.15	0.043*
nFmax (g.u.)	78.33 ± 39.48	59.49 ± 22.42	0.030*	69.64 ± 36.71	56.06 ± 28.21	0.062	81.99 ± 42.97	61.25 ± 23.03	0.036*	73.30 ± 37.64	57.69 ± 27.16	0.048*	65.74 ± 35.13	53.10 ± 26.04	0.079

### Heatmaps

**Figure 5:**
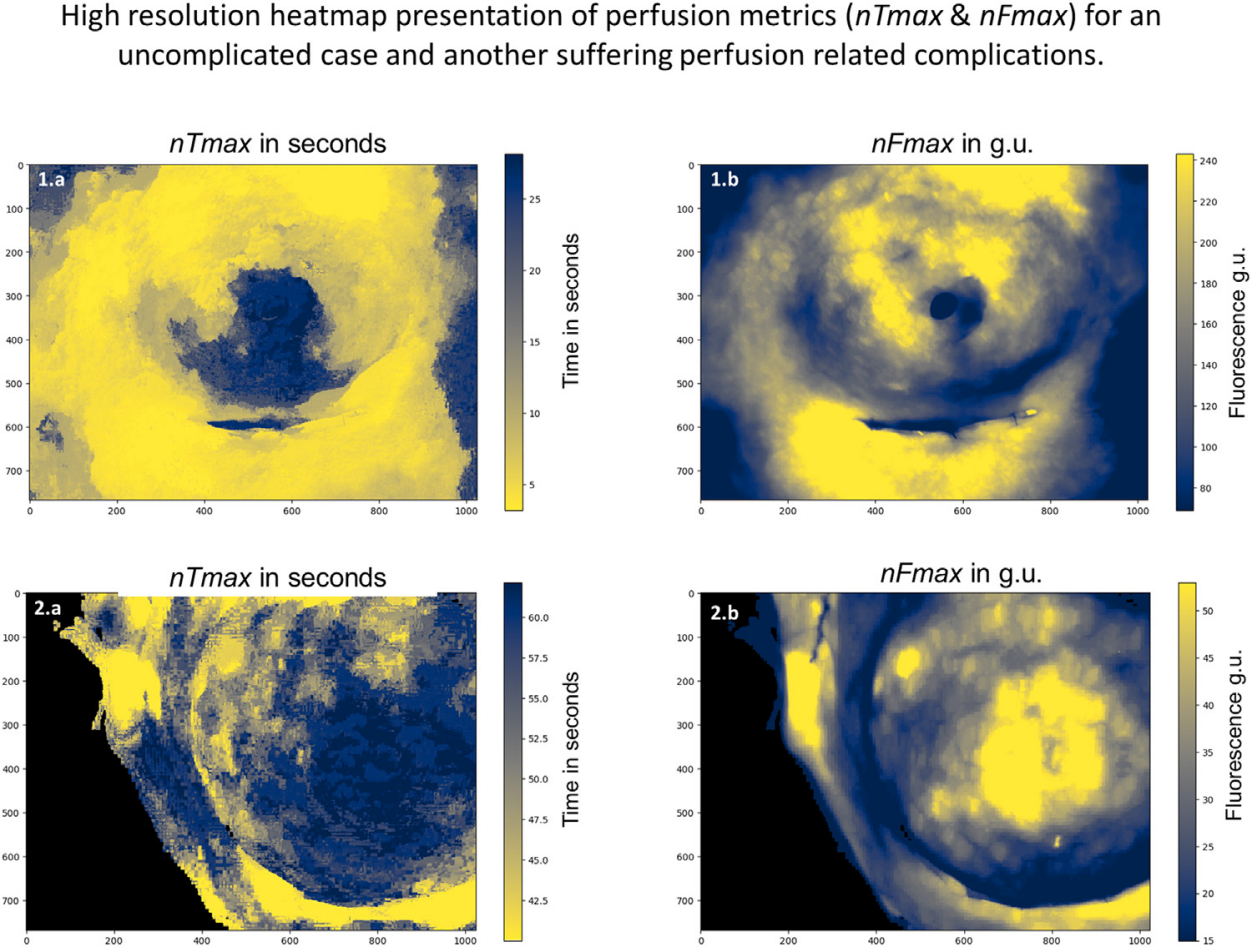
High-resolution heatmaps (per pixel) of Q-ICGFA metrics (*nF_max_* and *nT_max_*). x and y-axes denote the coordinates on the image, and the colour bar on the far right denotes the value the colour represents. Yellow denotes better perfusion metrics, i.e., brighter *nF_max_* and faster *nT_max_* (versus blue). Top heatmaps (1.a and b) from an angiogram that follows NSM for DCIS (48-year-old lady) who had an uneventful recovery post tissue expander insertion. Bottom heatmaps (2.a and b) from an angiogram that follows unilateral right NSM and axillary clearance (47-year-old, T2N1). The patient required debridement for an infected wound, but the expander was successfully preserved. N.B. scales display different ranges, e.g., top heatmap (*nF_max_*, no PRC) although darker displays a higher range than below (80-240 versus 15-50 g.u.).

Regarding the patient recordings selected for high-resolution heatmaps (n=6), fluorescence time series were extracted at 786432 pixels per frame, creating visual representations of the dynamic metrics (see Figure 5 for two illustrative cases).

### ML

**Figure 6:**
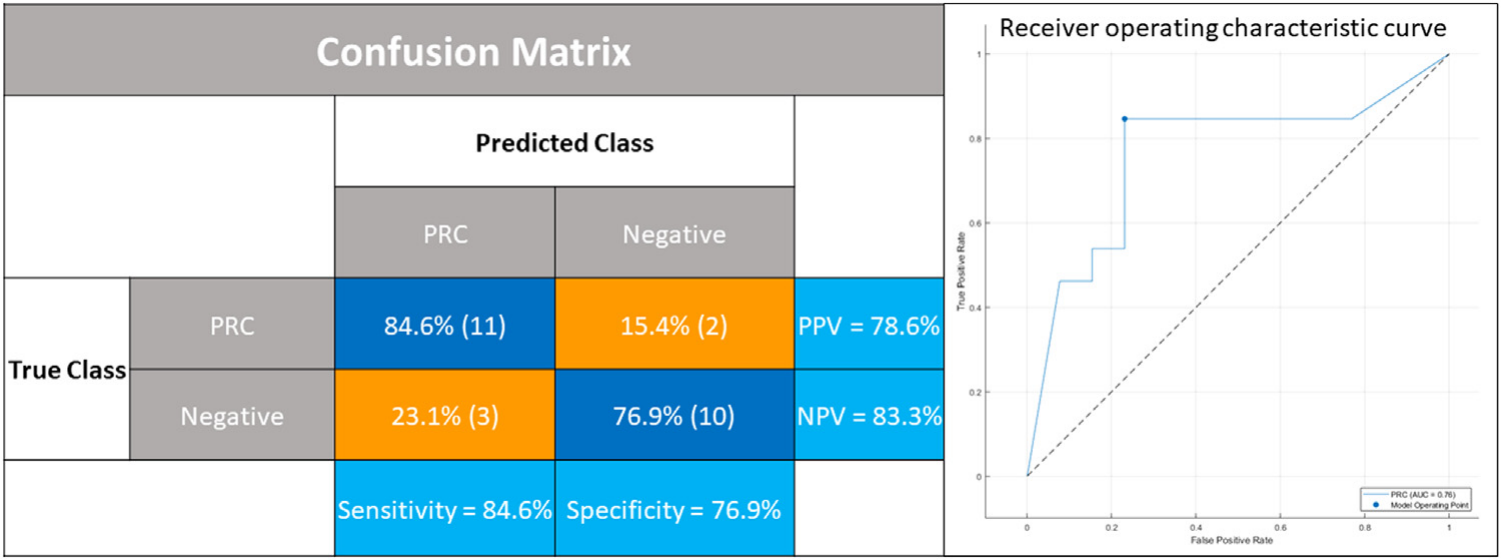
Confusion matrix on the left for the naïve Bayes machine learning model trained on the fluorescence metrics of a balanced data subset (n=26). PRC denotes perfusion-related complications, PPV positive predictive value, and NPV negative predictive value. The receiver operating characteristic (ROC) curve for the model is on the right. AUC denotes the area under the ROC curve.

The *nT_max_* and *peak time* of the inner third concentric region were ranked as the most predictive for PRC. The naïve Bayes ML algorithm (refined over 30 iterations) proved most accurate in predicting PRC with a sensitivity of 84.6%, specificity of 76.9%, PPV of 84.6%, NPV of 76.9%, and an overall accuracy of 80.77%. The area AUC was 0.76 (see Figure 6).

## Discussion

Risk factors for complications following NSM and IBR include radiotherapy (previous or adjuvant),^21^^,^^22^ raised BMI (30 kg/m^2^, which also implies larger mastectomy flaps^23^), diabetes mellitus, smoking, thin mastectomy flaps (<8 mm)^24^ as well as perhaps age and chemotherapy.^23^^,^^25^ As poor tissue perfusion intraoperatively is a major contributor to complications, SIBR seeks to bridge the gap between the benefits of IBR and the lower complication rates of delayed reconstructions by selectively delaying reconstruction temporarily in those suspected of poor flap perfusion to allow perfusion to improve.^6^ SIBR portends to expand the scope of NSM and IBR to higher-risk patients on an individualised basis, including those with larger and ptotic breasts.^26^

Previously investigated ICGFA-based surgical guidance has involved subjective interpretation with only point-in-time fluorescence intensity quantification from static ICGFA frames utilising software that measures absolute values or relative percentages between two regions.^27^^,^^28^ However, this strategy underutilises the data available both across the video's entire field of view and time during ICGFA. Applying quantification to the full fluorescence time series has the potential to develop better-informed surgical recommenders to stratify patients and hopefully improve outcomes post-NSM reconstruction (and indeed potentially other implant/autologous-based reconstruction). Such tools may support risk stratification (and SIBR) or they may impact factors such as the rate, timing, technique, stages, and costs (e.g. ADM use^29^) of reconstruction.

In the previous clinical study from which this dataset was generated,^12^ breast reconstruction was deferred in patients with poor perfusion by current clinical and ICGFA criteria, with a significant improvement in perfusion being evident at the second assessment a week later. In this current work, we confirm differences in initial brightness in deferred patients, with significant improvements in intensity and chronology at the second assessment. However, we also find distinct Q-ICGFA curves in cases developing PRC, with diminished brightness and slower onset even within the cohort of patients clinically deemed to have satisfactory perfusion in the original study. Our feature ranking has also identified flow patterns in the centre of the flap (including the NAC) as the most prognostic and has implications for guiding visual ICGFA interpretation. However, as these dynamic changes can be difficult to fully appreciate visually, a heatmap display of relevant metrics (as shown feasible here) may be useful, although the determination of decision-triggering thresholds requires further investigation.

Our ML model computed a sensitivity and specificity of 84.6% and 76.9%, respectively, with an AUC of 0.763, which is encouraging for exploratory work.^30^ The model correctly predicted complications in 11 of 13 patients, which is especially notable given that this group had already been filtered by the best current clinical judgement. This model was developed on explainable mechanistic biophysics and statistical associations.^31^ Other clinical applications of AI^32^ used deep learning. This type of AI requires training with thousands of still images and is less explainable, with concerns of unpredictable bias. Predictive tools have also been trained on patient demographics,^33^ and these could potentially be pooled with fluorescence data to train better models.

This dataset features a real-world series of cases; however, it has limitations, most obviously as a retrospective analysis and as a result of the original study design (which was an open-label case series so risking allocation bias and lacking a control group). Although significant associations were demonstrated, other inferences may have been missed due to type two error, as the cohort was not prospectively powered. This research did show improved perfusion following the delay period but has not identified the causative physiological changes. Furthermore, complications were associated with Q-ICGFA parameters from all reconstructions (including both IBR and SIBR). While this supported generalisable learnings, this heterogeneity may have impacted these correlations. Also, mastectomy flap thickness could not be standardised as this is dictated by anatomical planes, which can vary in depth between patients.

Video attrition could also have impacted cohort demographics, although the overall PRC rate is similar to other ICGFA case series.^10^ Chest wall movement with respiration and movements associated with holding the imager by hand generate artefacts. While here mathematical smoothing addressed fine motion artefacts, this does result in data loss, and also, statistical associations were not consistently significant for both smoothed and non-smoothed metrics. Non-smoothed metrics did, in fact, demonstrate greater predictive capabilities in feature ranking than smoothed ones. Potential stabilisation strategies include mounting the camera on a frame or computational *post hoc* video processing.^34^ Image saturation occurred when the fluorescence exceeded the camera's maximum detectable intensity, thus precluding discrimination of dynamic signal variations. This can be addressed potentially by using lower doses of ICG. A stronger clinical protocol could also offset other problems, such as bleeding (addressable by fastidious haemostasis), to maximise patient inclusion.

The fluorescence metrics used have been biochemically correlated to malperfusion in other tissue types^35^, but not breast skin or the NAC. Also, segregation of a separate testing and validation cohort was not numerically possible, limiting optimisation and generalisability of the ML model, which still needs validation on an unseen dataset and ideally in a prospective clinical trial. The ML model was trained to detect PRC within 90 days, which is a composite outcome. The selection of PRC, including surgical site infections (which may also map to smoking status and previous history of irradiation^36^) as a compound metric, collects relevant outcomes into a single binary predictor as has been used previously in other studies involving ICGFA.^10^ Other classification systems, which subclassify complications, such as the Dindo–Clavien classification^37^, would have further subdivided the data, complicating ML training due to lack of statistical power and data imbalance.

Our dataset was unbalanced, as the patients without PRC (88.8%) greatly outnumbered those who did not. This means that simple accuracy-directed models would be correct 88.8% of the time when they never predict PRC but with no sensitivity. Addressing such imbalance is acknowledged as necessary in the training of surgical ML where complications occur in a minority of patients.^38^^,^^39^ Alternative clinically applied data balancing strategies include *over-sampling* by duplicating the *minority class* to match the *majority* class (which is prone to overfitting) or supplementing the *minority* class with synthetic data.^40^

While this work focused on NSM mostly via an inframammary incision, its methods could be applied to other operation types. However, videos lacking NAC would introduce heterogeneity in the analysis and ML, and there is computational difficulty in differentiating gaps in central incisions (which would not fluoresce) from malperfused tissue (see Figure 4.3). This could be addressed via computer vision, especially if a white light image feed could be concomitantly presented. Although the system used here has some advantages (including a standardised target-distance camera distance via a laser marker), other cameras have a wider field of view, permitting simultaneous bilateral breast imaging.^41^ This capability would also allow inclusion of a normal area of skin which could help standardise computerised interpretations by accounting for ICG pharmacodynamics differences between patients (including those due to physiology e.g., blood protein levels^42^).

## Conclusions

In summary, computational assessment of post-NSM ICGFA identifies dynamic perfusion patterns across the mastectomy flap with regional associations with post-reconstruction PRC, even among those patients who had passed the best current surgical interrogations in this retrospective series. There is an opportunity for further technological development, and with further validation (which now can be planned with this proof of concept now established), such methods could guide surgeons better in their intraoperative decision-making.

## Contributions

Dr J Dalli and Dr C L Nguyen should be recognised as joint first authors*. All authors contributed to the study design, analysis, and drafting of the manuscript.

## Declaration of competing interest

Professor Ronan Ambrose Cahill is named on a patent filed in relation to processes for visual determination of tissue biology, receives speaker fees from Stryker Corp, Ethicon/J&J and Olympus, research funding from Intuitive Corp, consultancy fees from Arthrex, Diagnostic Green, Distalmotion and Medtronic (Touch Surgery) and holds research funding from the Irish Government (DTIF) in collaboration with IBM Research in Ireland, from EU Horizon 2020 in collaboration with Palliare and Steripak, from Horizon Europe in collaboration with Arctur, and from Intuitive and Medtronic for specific research and development awards.

Drs Jeffrey Dalli and Niall Phillip Hardy were employed as researchers in the DTIF. Dr Jeffrey Dalli is a recipient of the TESS (Malta) Scholarship and is named on a patent filed by University College Dublin concerning technologies related to tissue perfusion.

Drs Jonathan P Epperlein is a full-time employee of IBM Research, a division of IBM, which provides technical products and services worldwide to government, healthcare, and life-sciences companies. He is named on filed and granted patents concerning technologies related to the subject matter of this paper.

Mr A Jindal and Doctors C L Nguyen, C Pulitano, and S Warrier report no disclosures.

## Appendix Supplementary materials

Image, application 1

## References

[1] Neuburger J, MacNeill F, Jeevan R, van der Meulen JHP, Cromwell DA. (2013). Trends in the use of bilateral mastectomy in England from 2002 to 2011: retrospective analysis of hospital episode statistics. BMJ Open.

[2] Senkus E, Kyriakides S, Ohno S (2015). Primary breast cancer: ESMO Clinical Practice Guidelines for diagnosis, treatment and follow-up. Ann Oncol.

[3] Agha RA, Al Omran Y, Wellstead G (2019). Systematic review of therapeutic nipple-sparing versus skin-sparing mastectomy. BJS Open.

[4] Al-Ghazal S, Sully L, Fallowfield L, Blamey R. (2000). The psychological impact of immediate rather than delayed breast reconstruction. Eur J Surg Oncol.

[5] Van Bommel ACM, De Ligt KM, Schreuder K (2020). The added value of immediate breast reconstruction to health-related quality of life of breast cancer patients. Eur J Surg Oncol.

[6] Yoon AP, Qi J, Brown DL (2018). Outcomes of immediate versus delayed breast reconstruction: results of a multicenter prospective study. The Breast.

[7] Wang M, Huang J, Chagpar AB. (2020). Is nipple sparing mastectomy associated with increased complications, readmission and length of stay compared to skin sparing mastectomy?. Am J Surg.

[8] Radovanovic Z, Radovanovic D, Golubovic A (2010). Early complications after nipple-sparing mastectomy and immediate breast reconstruction with silicone prosthesis: results of 214 procedures. Scand J Surg.

[9] Kobraei EM, Nimtz J, Wong L (2012). Risk factors for adverse outcome following skin-sparing mastectomy and immediate prosthetic reconstruction. Plast Reconstr Surg.

[10] Lauritzen E, Damsgaard TE. (2021). Use of indocyanine green angiography decreases the risk of complications in autologous- and implant-based breast reconstruction: a systematic review and meta-analysis. J Plast Reconstr Aesthet Surg.

[11] Nguyen CL, Dayaratna N, Comerfield AP (2022). Cost-effectiveness of indocyanine green angiography in postmastectomy breast reconstruction. J Plast Reconstr Aesthet Surg.

[12] Nguyen CL, Liu YHA, Lata T (2022). Utility of indocyanine green angiography in delaying breast reconstruction postmastectomy. Eur J Plast Surg.

[13] Nguyen CL, Tam SKM, Easwaralingam N (2022). Patterns of ischaemia and reperfusion in nipple-sparing mastectomy reconstruction with indocyanine green angiography. J Plast Reconstr Aesthet Surg.

[14] Pruimboom T, Schols RM, Van Kuijk SM, Van der Hulst RR, Qiu SS. (2020). Indocyanine green angiography for preventing postoperative mastectomy skin flap necrosis in immediate breast reconstruction. Cochrane Database Syst Rev.

[15] Hardy NP, Dalli J, Khan MF (2021). Inter-user variation in the interpretation of near infrared perfusion imaging using indocyanine green in colorectal surgery. Surg Endosc.

[16] Epperlein JP, Hardy NP, Aonghusa PM, Cahill RA. Extracting, visualizing, and learning from dynamic data: perfusion in surgical video for tissue characterization. 2022.

[17] Lütken CD, Achiam MP, Osterkamp J, Svendsen MB, Nerup N. (2021). Quantification of fluorescence angiography: toward a reliable intraoperative assessment of tissue perfusion - a narrative review. Langenbecks Arch Surg.

[18] Park S-H, Park H-M, Baek K-R (2020). Artificial intelligence based real-time microcirculation analysis system for laparoscopic colorectal surgery. World J Gastroenterol.

[19] Warrier S, Nguyen CL, Easwaralingam N. (2021). Acellular dermal matrices in breast reconstruction: a narrative review and institutional perspective. Ann Breast Surg.

[20] Liu XY, Wu J, Zhou ZH. (2009). Exploratory undersampling for class-imbalance learning. IEEE Trans Syst, Man, Cybern, Part B.

[21] Chetta MD, Aliu O, Zhong L (2017). Reconstruction of the irradiated breast. Plast Reconstr Surg.

[22] Kell MR, Barry M. (2013). Effects of post-mastectomy radiotherapy on breast reconstruction. BMJ : Br Med J.

[23] Hölmich LR, Sayegh F, Salzberg CA. (2023). Immediate or delayed breast reconstruction: the aspects of timing, a narrative review. Ann Breast Surg.

[24] Frey JD, Salibian AA, Choi M, Karp NS. (2017). Mastectomy flap thickness and complications in nipple-sparing mastectomy: objective evaluation using magnetic resonance imaging. Plast Reconstr Surg Glob Open.

[25] Reintgen C, Leavitt A, Pace E, Molas-Pierson J, Mast BA. (2016). Risk factor analysis for mastectomy skin flap necrosis: implications for intraoperative vascular analysis. Ann Plast Surg.

[26] Zenn MR. (2015). Staged immediate breast reconstruction. Plast Reconstr Surg.

[27] Munabi NC, Olorunnipa OB, Goltsman D, Rohde CH, Ascherman JA. (2014). The ability of intra-operative perfusion mapping with laser-assisted indocyanine green angiography to predict mastectomy flap necrosis in breast reconstruction: a prospective trial. J Plast Reconstr Aesthet Surg.

[28] Phillips BT, Lanier ST, Conkling N (2012). Intraoperative perfusion techniques can accurately predict mastectomy skin flap necrosis in breast reconstruction: results of a prospective trial. Plast Reconstr Surg.

[29] Wagner DS, Mirhaidari SJ. (2019). Capsulectomy, implant exchange, and placement of acellular dermal matrix is effective in treating capsular contracture in breast augmentation patients. Aesthet Surg J.

[30] Mandrekar JN. (2010). Receiver operating characteristic curve in diagnostic test assessment. J Thoracic Oncol.

[31] Hardy NP, Dalli J, Mac Aonghusa P, Neary PM, Cahill RA (2021). Biophysics inspired artificial intelligence for colorectal cancer characterization. Artif Intell Gastroenterol.

[32] McKinney SM, Sieniek M, Godbole V (2020). International evaluation of an AI system for breast cancer screening. Nature.

[33] Hassan AM, Biaggi AP, Asaad M, et al. Development and assessment of machine learning models for individualized risk assessment of mastectomy skin flap necrosis. Ann Surg 9000. 10.1097/SLA.0000000000005386 35129476

[34] Goshtasby A. (1986). Piecewise linear mapping functions for image registration. Pattern Recognit.

[35] Diana M, Agnus V, Halvax P (2015). Intraoperative fluorescence-based enhanced reality laparoscopic real-time imaging to assess bowel perfusion at the anastomotic site in an experimental model. Br J Surg.

[36] Olsen MA, Lefta M, Dietz JR (2008). Risk factors for surgical site infection after major breast operation. J Am Coll Surg.

[37] Dindo D, Demartines N, Clavien PA. (2004). Classification of surgical complications: a new proposal with evaluation in a cohort of 6336 patients and results of a survey. Ann Surg.

[38] Liu X, Guo L, Wang H (2022). Research on imbalance machine learning methods for MRT1WI soft tissue sarcoma data. BMC Med Imaging.

[39] Stam WT, Goedknegt LK, Ingwersen EW (2022). The prediction of surgical complications using artificial intelligence in patients undergoing major abdominal surgery: a systematic review. Surgery.

[40] Bach M, Werner A, Żywiec J, Pluskiewicz W. (2017). The study of under- and over-sampling methods’ utility in analysis of highly imbalanced data on osteoporosis. Inf Sci.

[41] Dalli J, Jindal A, Gallagher G (2023). Evaluating clinical near-infrared surgical camera systems with a view to optimizing operator and computational signal analysis. J Biomed Opt.

[42] Kim GY, Bae KS, Noh GJ, Min WK. (2009). Estimation of indocyanine green elimination rate constant k and retention rate at 15 min using patient age, weight, bilirubin, and albumin. J Hepatobiliary Pancreat Surg.

