# Supplementary material for: A feasibility study assessing quantitative indocyanine green angiographic predictors of reconstructive complications following nipple-sparing mastectomy
Source: JPRAS Open. 2024 Jan 26;40:32–47. doi: 10.1016/j.jpra.2024.01.012 (PMC10904167; doi:10.1016/j.jpra.2024.01.012)
Supplement: Supplementary file 1 [file mmc1.docx]

Supplementary table 1. Additional metrics to those reported in table 3 Fluorescence metrics for those undergoing IBR were compared to delayed cases at the index (first) procedure via Mann-Whitney U test. This table shows the mean extracted quantitative fluorescence metrics relating to intensity in g.u. and chronology in seconds for the annotated breast and divided by quadrants and concentric regions. Features were extracted with a curve detection algorithm following mathematical smoothing. Significant results are marked with *.

| **Supplementary 1 (additional data to table 3): Fluorescence metrics at the first (index) procedure for IBR versus delayed cases** | | | | | | | | | | | | | | | |
| --- | --- | --- | --- | --- | --- | --- | --- | --- | --- | --- | --- | --- | --- | --- | --- |
| Quadrant | Whole Breast | | | Lower Lateral | | | Lower Medial | | | Upper Lateral | | | Upper Medial | | |
|  | IBR | Delay | p | IBR | Delay | p | IBR | Delay | p | IBR | Delay | p | IBR | Delay | p |
| intensity at latency (g.u.) | 3.18 ± 3.72 | 2.89 ± 5.64 | 0.067 | 3.24 ± 4.05 | 3.14 ± 6.24 | 0.177 | 3.24 ± 4.20 | 2.76 ± 5.07 | 0.078 | 3.08 ± 3.68 | 2.89 ± 5.90 | 0.138 | 3.16 ± 3.66 | 2.66 ± 4.89 | 0.039* |
| T50 (s) | 12.65 ± 4.74 | 11.91 ± 4.59 | 0.488 | 12.68 ± 4.26 | 13.87 ± 6.35 | 0.764 | 12.25 ± 5.37 | 13.60 ± 6.22 | 0.368 | 12.25 ± 5.60 | 12.86 ± 5.90 | 0.645 | 12.39 ± 5.22 | 12.71 ± 5.40 | 0.721 |
| F50 (g.u.) | 38.30 ± 15.84 | 30.08 ± 12.61 | 0.033* | 36.15 ± 14.43 | 27.60 ± 15.41 | 0.013* | 42.18 ± 18.50 | 29.45 ± 13.44 | 0.005* | 39.88 ± 18.42 | 33.66 ± 19.03 | 0.145 | 43.76 ± 21.55 | 37.06 ± 17.43 | 0.344 |
| Upslope50 (g.u./s) | 3.50 ± 3.01 | 2.73 ± 1.70 | 0.428 | 3.08 ± 2.26 | 2.31 ± 1.88 | 0.091 | 4.31 ± 3.95 | 2.62 ± 1.84 | 0.058 | 3.93 ± 3.53 | 3.08 ± 2.29 | 0.340 | 4.43 ± 4.22 | 3.79 ± 3.98 | 0.399 |
| Concentric Zones | Inner half | | | Outer half | | | Inner third | | | Middle third | | | Outer third | | |
|  | IBR | Delay | p | IBR | Delay | p | IBR | Delay | p | IBR | Delay | p | IBR | Delay | p |
| intensity at latency (g.u.) | 3.51 ± 4.44 | 3.32 ± 7.10 | 0.118 | 2.89 ± 3.14 | 2.47 ± 4.16 | 0.030* | 3.74 ± 4.92 | 3.73 ± 8.48 | 0.326 | 3.12 ± 3.63 | 2.72 ± 4.99 | 0.064 | 2.71 ± 2.84 | 2.38 ± 3.94 | 0.037* |
| T50 (s) | 14.02 ± 5.83 | 12.78 ± 7.01 | 0.220 | 11.97 ± 4.49 | 12.57 ± 5.54 | 0.918 | 14.62 ± 6.50 | 17.16 ± 11.35 | 0.654 | 12.38 ± 5.01 | 11.30 ± 4.52 | 0.288 | 11.55 ± 4.39 | 12.13 ± 4.83 | 0.567 |
| F50 (g.u.) | 43.31 ± 16.41 | 30.84 ± 13.65 | 0.001* | 35.49 ± 15.88 | 28.33 ± 14.15 | 0.039* | 45.90 ± 18.36 | 27.01 ± 17.93 | <0.001* | 38.66 ± 16.53 | 31.06 ± 12.92 | 0.076 | 32.94 ± 14.89 | 27.00 ± 14.42 | 0.037* |
| Upslope50 (g.u./s) | 3.62 ± 2.84 | 2.60 ± 1.58 | 0.222 | 3.52 ± 3.32 | 2.69 ± 2.01 | 0.263 | 3.66 ± 2.81 | 2.10 ± 1.84 | 0.006* | 3.67 ± 3.27 | 2.96 ± 1.74 | 0.694 | 3.37 ± 3.09 | 2.64 ± 2.13 | 0.237 |

Supplementary table 2. Additional metrics to those reported in table 4 Fluorescence metrics for those patients who were delayed at first assessment versus the same cases at the second assessment a week later via Wilcoxon-signed rank. This table shows the mean extracted quantitative fluorescence metrics relating to intensity in g.u. and chronology in seconds for the annotated breast and divided by quadrants and concentric regions. Features were extracted with a curve detection algorithm following mathematical smoothing. Significant results are marked with *.

| **Supplementary 2 (additional data to table 4): Fluorescence metrics for delayed cases at first assessment versus second assessment a week later** | | | | | | | | | | | | | | | |
| --- | --- | --- | --- | --- | --- | --- | --- | --- | --- | --- | --- | --- | --- | --- | --- |
| Quadrant | Whole Breast | | | Lower Lateral | | | Lower Medial | | | Upper Lateral | | | Upper Medial | | |
|  | First | Second | P | First | Second | p | First | Second | p | First | Second | p | First | Second | p |
| intensity at latency (g.u.) | 3.73 ± 7.38 | 2.46 ± 1.50 | 0.026* | 3.95 ± 7.99 | 2.58 ± 1.66 | 0.021* | 3.54 ± 6.66 | 2.49 ± 1.56 | 0.015* | 3.80 ± 7.78 | 2.21 ± 1.25 | 0.039* | 3.42 ± 6.43 | 2.53 ± 2.23 | 0.016* |
| T50 (s) | 11.53 ± 5.02 | 9.98 ± 3.11 | 0.221 | 14.50 ± 7.40 | 10.97 ± 4.20 | 0.209 | 13.33 ± 6.49 | 9.69 ± 3.35 | 0.071 | 12.57 ± 6.00 | 9.95 ± 3.35 | 0.074 | 11.63 ± 4.72 | 9.31 ± 3.02 | 0.112 |
| F50 (g.u.) | 29.30 ± 10.31 | 55.89 ± 19.91 | 0.001* | 26.11 ± 13.94 | 51.55 ± 19.10 | 0.015* | 30.61 ± 13.38 | 55.47 ± 20.73 | 0.006* | 31.38 ± 14.86 | 57.74 ± 20.89 | 0.011* | 38.35 ± 12.39 | 60.87 ± 23.34 | 0.004* |
| Upslope50 (g.u./s) | 2.67 ± 1.23 | 5.82 ± 2.69 | 0.003* | 2.24 ± 1.82 | 5.30 ± 3.02 | 0.015* | 2.69 ± 1.75 | 5.89 ± 2.42 | 0.019* | 2.96 ± 2.18 | 6.27 ± 3.33 | 0.003* | 3.64 ± 2.18 | 6.81 ± 3.62 | 0.027* |
| Concentric Zones | Inner half | | | Outer half | | | Inner third | | | Middle third | | | Outer third | | |
|  | First | Second | p | First | Second | p | First | Second | p | First | Second | p | First | Second | p |
| intensity at latency (g.u.) | 4.34 ± 9.27 | 2.52 ± 1.48 | 0.031* | 3.11 ± 5.47 | 2.41 ± 1.55 | 0.031* | 4.91 ± 11.00 | 2.53 ± 1.52 | 0.042* | 3.46 ± 6.54 | 2.48 ± 1.56 | 0.031* | 3.01 ± 5.18 | 2.29 ± 1.46 | 0.033* |
| T50 (s) | 12.56 ± 7.99 | 10.17 ± 2.89 | 0.476 | 11.52 ± 5.32 | 9.68 ± 3.17 | 0.079 | 19.13 ± 13.62 | 10.87 ± 3.08 | 0.028* | 10.75 ± 4.99 | 10.08 ± 3.26 | 0.300 | 11.48 ± 4.79 | 9.49 ± 3.19 | 0.057 |
| F50 (g.u.) | 28.68 ± 12.20 | 57.96 ± 21.01 | 0.003* | 28.29 ± 10.11 | 53.77 ± 20.21 | <0.001* | 26.16 ± 15.40 | 59.62 ± 22.60 | 0.003* | 30.11 ± 10.25 | 56.40 ± 20.36 | <0.001* | 25.75 ± 10.93 | 50.48 ± 19.30 | <0.001* |
| Upslope50 (g.u./s) | 2.48 ± 1.13 | 5.87 ± 2.75 | 0.008* | 2.76 ± 1.52 | 5.85 ± 2.95 | 0.001* | 1.84 ± 1.27 | 5.70 ± 2.93 | 0.003* | 2.93 ± 1.31 | 5.86 ± 2.86 | 0.003* | 2.51 ± 1.52 | 5.67 ± 2.96 | <0.001* |

Supplementary table 3. Additional metrics to those reported in table 5. Fluorescence metrics for patients who underwent reconstruction (IBR and SIBR) and suffered PRC were compared to those who did not via Mann-Whitney U test. This table shows the mean extracted quantitative fluorescence metrics relating to intensity in g.u. and chronology in seconds for the annotated breast and divided by quadrants and concentric regions. Features were extracted with a curve detection algorithm following mathematical smoothing. Significant results are marked with *.

| **Supplementary 3 (additional data to table 5): Fluorescence metrics for patients not suffering PRC versus does who did for all reconstructed cases (IBR and SIBR)** | | | | | | | | | | | | | | | | |
| --- | --- | --- | --- | --- | --- | --- | --- | --- | --- | --- | --- | --- | --- | --- | --- | --- |
| Quadrant | Whole Breast | | | Lower Lateral | | | Lower Medial | | | Upper Lateral | | | Upper Medial | | | |
|  | No PRC | PRC | p | No PRC | PRC | p | No PRC | PRC | p | No PRC | PRC | p | No PRC | PRC | p |  |
| intensity at latency (g.u.) | 2.67 ± 3.25 | 5.08 ± 7.28 | 0.682 | 2.78 ± 3.80 | 5.18 ± 7.25 | 0.324 | 2.66 ± 3.39 | 5.18 ± 7.18 | 0.611 | 2.55 ± 3.10 | 5.08 ± 7.99 | 0.331 | 2.64 ± 3.17 | 4.71 ± 6.58 | 0.523 |  |
| T50 (s) | 11.47 ± 4.34 | 14.06 ± 5.49 | 0.073 | 12.05 ± 4.54 | 14.57 ± 6.92 | 0.293 | 11.65 ± 5.29 | 14.20 ± 5.59 | 0.072 | 11.67 ± 5.21 | 12.90 ± 6.89 | 0.593 | 11.58 ± 5.02 | 13.63 ± 5.49 | 0.147 |  |
| F50 (g.u.) | 40.89 ± 18.67 | 31.34 ± 12.67 | 0.027* | 38.27 ± 18.82 | 31.44 ± 11.15 | 0.203 | 42.77 ± 20.13 | 33.35 ± 14.42 | 0.100 | 42.82 ± 21.09 | 33.59 ± 16.11 | 0.070 | 46.20 ± 23.38 | 37.49 ± 15.97 | 0.148 |  |
| Upslope50 (g.u./s) | 4.09 ± 3.33 | 2.45 ± 1.85 | 0.010* | 3.64 ± 3.16 | 2.71 ± 2.38 | 0.156 | 4.47 ± 3.58 | 2.73 ± 2.29 | 0.027* | 4.45 ± 3.91 | 3.31 ± 3.05 | 0.105 | 5.04 ± 4.65 | 3.18 ± 2.74 | 0.066 |  |
| Concentric Zones | Inner half | | | Outer half | | | Inner third | | | Middle third | | | Outer third | | | |
|  | No PRC | PRC | p | No PRC | PRC | p | No PRC | PRC | p | No PRC | PRC | p | No PRC | PRC | p |  |
| intensity at latency (g.u.) | 2.92 ± 4.04 | 5.79 ± 8.75 | 0.550 | 2.44 ± 2.62 | 4.35 ± 5.74 | 0.705 | 3.11 ± 4.75 | 6.36 ± 9.80 | 0.467 | 2.62 ± 3.07 | 4.82 ± 6.71 | 0.674 | 2.31 ± 2.40 | 4.14 ± 5.35 | 0.611 |  |
| T50 (s) | 12.63 ± 5.84 | 14.64 ± 5.90 | 0.170 | 11.41 ± 4.70 | 13.05 ± 4.59 | 0.124 | 13.94 ± 7.79 | 16.98 ± 7.24 | 0.052 | 11.41 ± 4.76 | 12.62 ± 4.53 | 0.261 | 11.12 ± 4.46 | 12.08 ± 4.08 | 0.312 |  |
| F50 (g.u.) | 44.34 ± 19.92 | 35.17 ± 11.00 | 0.112 | 37.34 ± 18.63 | 30.83 ± 14.84 | 0.102 | 45.34 ± 22.94 | 32.96 ± 12.70 | 0.044* | 41.03 ± 19.03 | 32.80 ± 13.93 | 0.071 | 35.01 ± 17.80 | 29.13 ± 13.79 | 0.129 |  |
| Upslope50 (g.u./s) | 4.13 ± 3.29 | 2.56 ± 1.68 | 0.051 | 3.94 ± 3.49 | 2.63 ± 2.25 | 0.060 | 4.03 ± 3.35 | 2.12 ± 1.55 | 0.015* | 4.20 ± 3.49 | 2.75 ± 2.03 | 0.037* | 3.78 ± 3.29 | 2.70 ± 2.43 | 0.102 |  |
